# Information Sharing in the School Setting During a Public Health Emergency

**DOI:** 10.1177/1942602X20925031

**Published:** 2020-05-15

**Authors:** Christina Baker, Cynthia A. Galemore, Kerri McGowan Lowrey

**Affiliations:** Christina Baker, MS, NCSN, RN-BC, School Health Consultant, Children’s Hospital Colorado, Aurora, CO; Cynthia A. Galemore, MSEd, BSN, RN, NCSN, FNASN, Independent School Health Consultant, Olathe, KS; Kerri McGowan Lowrey, JD, MPH, Deputy Director and Director for Grants and Research, Network for Public Health Law, Eastern Region, University of Maryland Francis King Carey School of Law, Baltimore, MD

**Keywords:** HIPAA, FERPA, public health emergencies, COVID-19, coronavirus, schools, school nursing

## Abstract

The Family Educational Rights and Privacy Act of 1974 is the federal law that protects the privacy of personally identifiable information from student education records and applies to all education entities that receive funding under any program administered by the U.S. Department of Education. The Health Insurance Portability and Accountability Act of 1996 is the federal law that establishes privacy requirements for patients’ protected health information. Together these privacy laws establish rules that guide school nurses in the sharing of student information, even in times of public health emergencies. The U.S. Department of Education and the U.S. Department of Health and Human Services have issued special updates to privacy laws in response to the Novel Coronavirus Disease providing certain waivers of typical privacy requirements and direction to allow the sharing of information during this public health emergency. The purpose of this article is to briefly review the privacy laws as they relate to schools, as well as to provide an overview of the recent waivers to assist school nurses, school administrators, healthcare professionals, and public health agencies in protecting the health and safety of students during this current public health emergency.

School nurses are the designated health professionals in the educational setting. As such, they must be educated in the nuances of the interplay between The Health Insurance Portability and Accountability Act of 1996 (HIPAA) and The Family Educational Rights and Privacy Act of 1974 (FERPA), especially in public health emergency situations. FERPA applies to educational agencies and institutions that are federally funded, and the HIPAA privacy rule applies to “covered entities” such as health plans, healthcare clearinghouses, and healthcare providers that transmit health information electronically (U.S. Department of Health and Human Services [DHHS] & U.S. Department of Education [DOE], 2019). Generally, school nurses are subject to FERPA, because the HIPAA Privacy Rule expressly *excludes* information from education records, including health information, from HIPAA’s definition of protected health information (PHI). “In short, when FERPA applies, HIPAA does not,” but there are circumstances when school nurses may be subject to HIPAA, such as if they practice in a private school that does not receive DOE funding and when they need to interact with HIPAA covered entities (Lowrey & Prechtel, 2019).

In a public health emergency, such as the Novel Coronavirus Disease (COVID-19) pandemic, FERPA and HIPAA rules still apply, but limited waivers and exceptions may allow for increased sharing of information. The National Association of School Nurses (NASN) provides a multitude of resources to members, such as continuing nursing education programs about data privacy laws (Lowrey & Prechtel, 2019) and information on the webpage (https://www.nasn.org/home). Another pertinent resource is the updated document titled Data Sharing Guidance for School Nurses (Lowrey, 2020). Table 1 provides an overall listing of school health record related COVID 19, HIPAA, and FERPA information and guidance from the Centers for Disease Control and Prevention, DHHS, DOE, and NASN.

**Table 1.** School Health Related COVID 19, HIPAA, and FERPA Resources

Organization	Type of information	Link/webpage
CDC	COVID-19: What to do if you are sick	https://www.cdc.gov/coronavirus/2019-ncov/if-you-are-sick/steps-when-sick.html
Lowrey (2020), Network for Public Health Law	Data sharing guidance for school nurses	https://www.networkforphl.org/wp-content/uploads/2020/01/Data-Sharing-Guidance-for-School-Nurses-with-Appendices-1-23-2020.pdf
Lowrey (2019a), Network for Public Health Law	Data privacy in school nursing (Part I)	https://www.networkforphl.org/wp-content/uploads/2019/12/Data-Privacy-in-School-Nursing-Navigating-the-Complex-Landscape-of-Data-Privacy-Laws-Part-1-1.pdf
Lowrey (2019b), Network for Public Health Law	Data privacy in school nursing (Part II)	https://www.networkforphl.org/wp-content/uploads/2020/01/Data-Privacy-in-School-Nursing-Part-II-1-23-2020.pdf
NASN	Coronavirus Disease 2019 resources	https://www.nasn.org/nasn/nasn-resources/practice-topics/covid19
U.S. Department Education	COVID-19 Resources	https://www.ed.gov/coronavirus
U.S. Department Health and Human Services	COVID-19 and HIPAA bulletin	https://www.hhs.gov/sites/default/files/hipaa-and-covid-19-limited-hipaa-waiver-bulletin-508.pdf
U.S. Department Education & U.S. Department Health and Human Services	Joint guidance: FERPA and HIPAA	https://www.hhs.gov/sites/default/files/2019-hipaa-ferpa-joint-guidance-508.pdf

*Note*. COVID-19 = Novel Coronavirus Disease-2019; HIPAA = The Health Insurance Portability and Accountability Act of 1996; FERPA = The Family Educational Rights and Privacy Act of 1974; CDC = Centers for Disease Control and Prevention; NASN = National Association of School Nurses.

## Implications for School Nurses

### FERPA

Generally, FERPA requires parental consent to disclose personally identifiable information (PII) from education records, including student health information, but the law does provide exceptions that allow school personnel to share certain PII without parental consent (34 CFR [Code of Federal Regulation] § 99. 31). For example, school nurses may share PII with appropriate officials in health or safety emergencies. In 2008, revised FERPA regulations were issued to clarify the “health or safety emergency exception,” which allows educational agencies and institutions to disclose PII from student education records, without prior written consent, to appropriate parties in the event of a health or safety emergency. “Appropriate parties” are those whose knowledge of the information is necessary to protect the health and safety of the student or other individuals (20 U.S.C.§ 1232g(b)(1)(I); 34 C.F.R. §§ 99.31(a)(10) and 99.36; Roberts & Zittoun, 2017; DHHS & DOE, 2019, p. 4). FERPA regulations originally required that these health and safety emergency provisions be “strictly construed,” but the 2008 revised rule removed the requirement for strict construction and instead allows schools, in making a determination about disclosing PII during emergencies, to take into account the totality of the circumstances related to a threat to the safety or health of the student or other individuals.

More recently, the DOE (2020) clearly outlined guidelines for the process of disclosing PII during a health emergency and emphasizes that disclosure decisions should be made on a case-by-case and rational basis. For example, disclosing PII to a public health department without prior consent is permissible when the school believes that the COVID-19 virus poses a serious risk to the health and safety of an individual student who attends their school. Similarly, each state has a list of reportable communicable diseases to which this consideration of health and safety applies. School nurses need to have a copy of the list and connect with local health departments/authorities as permitted and when applicable. Also, the student’s illness from COVID-19 and subsequent absence from school may be disclosed, without consent, to other students and their parents if that information is in a nonpersonally identifiable form. The disclosure must be done in a way “that does not disclose other information that, alone or in combination, would allow a reasonable person in the school community to identify the students who are absent due to COVID-19 with reasonable certainty” (DOE, 2020, p. 4).

### HIPAA

School nurses practicing in private or religious schools that do not accept federal funds (and also qualify as a covered entity) must comply with HIPAA. In response to the declaration of public health emergency in the face of COVID-19, DHHS Secretary Alex Azar has exercised his authority to waive sanctions and penalties against covered entities that do not comply with certain HIPAA provisions. Waivers that are possibly relevant to school nurses include the requirement to obtain a patient’s agreement to speak with family members or friends involved in the patient’s care; the requirement to distribute a notice of privacy practices; a patient’s right to request privacy restrictions; and the patient’s right to request confidential communications (DHHS, 2020). These waivers became effective on March 15, 2020.

Even without a waiver, HIPAA allows patient information to be shared without authorization in the following circumstances: (1) for treatment purposes, including permission for a school nurse practicing in a FERPA covered setting to request clarification of orders or recommendations from a student’s healthcare provider without parental consent (Gilsbach, 2017); (2) for public health activities, which would include most COVID-19 disclosures to public health authorities; (3) disclosures to family or other individuals identified by the patient as involved in the patient’s care “to prevent or lessen a serious or imminent threat to the health and safety of a person or the public” (DHHS, 2020, p. 3). Except for those made for treatment purposes, disclosures must always limit the information to the “minimum necessary” to accomplish the purpose, and covered entities must continue to implement reasonable safeguards against impermissible uses or disclosures (DHHS, 2020). For “treatment purposes” includes “the coordination or management of healthcare and related services by one or more healthcare providers and others, consultation between providers, and the referral of patients for treatment” (DHHS & DOE, 2019, p. 6).

### Communication During a Public Health Crisis

Larger school districts often have a dedicated position of a public information officer (PIO). School nurses should become knowledgeable about school policies and practices related to talking to the media, connecting media inquiries with the school’s PIO, communicable disease management, and the chain of command for placing health information in letters, newsletters, and on websites. In the absence of a dedicated PIO, school nurses should check with their immediate supervisor before releasing information except for what can be released to public health entities and allowable when speaking to a student’s individual healthcare provider. In addition, school nurses should partner with school administrators and PIOs to include a communication plan as part of a larger, crisis preparedness plan, in advance of disasters such as pandemics. This communication plan can provide guidance and steps for communication among staff, to the school community, and to the media and community at large. Frequent school nurse scenarios occurring during a communicable disease pandemic and appropriate responses are provided in Table 2.

**Table 2.** School Nurse Scenarios During a Communicable Disease Pandemic.

1. A parent calls the school nurse and reports that their child who attends the school has tested positive for COVID-19. What should the school nurse do regarding notification to the health department, the school administration, and the school community?
*Response*: Secretary of DHHS, Alex Azar, declared a public health emergency regarding COVID-19 on January 31, 2020. FERPA allows disclosure of student health information without parental consent to certain entities under the “health or safety emergency” exception. Even though a student’s positive COVID-19 test would be considered PII, the school nurse may report this information without parental consent to individuals whose knowledge of the information is necessary to protect the health or safety of students or other individuals (20 U.S.C. § 1232g(b)(1)(I); 34 C.F.R. §§ 99.31(a)(10) and 99.36; DOE, 2020). These may include public health officials, school administration, trained medical personnel, school staff, and parents. *• Public health departments*. Most state and local health departments are tracking COVID-19 infection throughout their jurisdictions. This surveillance directly informs public health and policy decisions. Knowledge of positive cases in specific schools enables health departments to track the origin of infection, identify others who may have been exposed, and make decisions accordingly to slow the spread of infection. *• School administration.* Work with school administration on refining business operation response plans and be involved in the planning process, which includes partnering with local public health authorities, updating infectious disease pandemic plans, and sharing essential communication strategies. Share the NASN resources such as the *Guidance for school principals and superintendents* and the *Pandemic flu checklist: K-12 school administrator* (Table 1). *• Outside medical personnel*. For student chronic disease management, obtain up-to-date information on clinic and hospital operations and how to best coordinate care in case of closures and restructuring services. *• School community-staff and parents*. Coordinate with your district’s PIO, or leadership, around communication of information and the technology platforms being used when schools are closed or operating online. Helpful resources such as NASN’s *Considerations for school nurses regarding care of students and staff that become ill at school or arrive sick* and the *ideas for school nurse activities during the COVID-19 pandemic* (see NASN website). Note that the “health or safety emergency” exception is limited in time to the period of the emergency and generally does not allow for a blanket release of PII from student education records.
2. A newspaper reporter calls the school nurse to ask if there are COVID 19 cases at the school (a student, teacher, or family member of someone in the school). What should the school nurse do?
*Response*: The school nurse should redirect the media to communicate with the district’s PIO or administration, who are responsible for communicating with the media, especially as related to a crisis. • FERPA only permits disclosures of PII under the health or safety emergency exception to “appropriate parties” whose knowledge of the information is necessary to protect the health or safety of students or other individuals. While the media plays an important role in keeping the community informed about an outbreak, they do not qualify as “appropriate parties” under FERPA’s health or safety emergency exception (DOE, 2020).
3. The school has a case of COVID-19, and the school nurse is concerned about a student in the same classroom with compromised immunity. What should the school nurse do?
*Response*: Working with the school administrator, the school should communicate to school staff and parents that there is a positive case of COVID-19 without using PII and in a manner that lessens the likelihood that others would be able to identify the COVID-positive student. The school nurse should also report the positive finding to the appropriate health department.
4. A student testing positive for COVID-19 returns to school ahead of what the school understands to be the required quarantine period. Can the school nurse call the student’s healthcare provider to discuss the required requirements for return to school?
*Response*: Yes. During public health emergencies, communications among healthcare providers are critical, and HIPAA and FERPA both allow for exchange of relevant information to protect students and others. • FERPA permits disclosures of PII under the health or safety emergency exception to “appropriate parties” whose knowledge of the information is necessary to protect the health or safety of students or other individuals. • Healthcare providers may share health information with a school nurse under HIPAA for “treatment purposes” without parent authorization (the so-called “provider-to-provider” exception under HIPAA). A pediatrician may freely discuss the student’s treatment record with the student’s school nurse. A school nurse may contact the physician to clarify the physician’s orders. In this scenario, clarification of the quarantine lifting date would constitute a permissible disclosure by the outside healthcare provider (Lowrey, 2019a, 2019b). • Some schools may qualify as HIPAA-covered entities if they do not accept funding under any program administered by the DOE. For these schools and other covered entities, the DHHS announced limited waivers of sanctions related to certain privacy provisions, such as the patient’s right to request confidential communications. See 45 CFR 164.522(b) and https://www.hipaajournal.com/hipaa-compliance-and-covid-19-coronavirus/. However, even without a waiver, the HIPAA Privacy Rule always allows patient information to be shared for treatment purposes and for public health activities. The HIPAA Privacy Rule recognizes the legitimate need for public health authorities and others responsible for ensuring public health and safety to have access to PHI that is necessary to carry out their public health mission. Therefore, the law permits covered entities to disclose PHI without individual authorization as necessary to prevent or control the spread of the disease or otherwise to carry out public health interventions or investigations. See 45 CFR 164.512(b)(1)(iv).

*Note*. COVID-19 = Novel Coronavirus Disease; DHHS = U.S. Department of Health and Human Services; FERPA = The Family Educational Rights and Privacy Act of 1974; PII = personally identifiable information; CFR = Code of Federal Regulation; DOE = U.S. Department of Education; NASN = National Association of School Nurses; PIO = public information officer; HIPAA = The Health Insurance Portability and Accountability Act of 1996; PHI = protected health information.

## Conclusion

Part of emergency preparedness is having a communication plan in place for pandemics, such as what is happening with COVID-19, including a plan for sharing allowed health information. COVID-19 shed light on the need for guidance in the event of public health emergencies, and certainly there will be other similar emergencies and infectious disease outbreaks in the future. It is prudent for school nurses to understand the privacy and information sharing requirements under both FERPA and HIPAA, as well as any waiver guidance to laws and regulations provided during health emergencies. School nurses must stay connected with public health authorities in their communities and counties, as well as with their professional organizations, for timely guidance to protect the health and safety of their students and school community.
